# Opening the “Black Box” Underlying Barriers to the Use of Canine Induced Pluripotent Stem Cells: A Narrative Review

**DOI:** 10.1089/scd.2022.0300

**Published:** 2023-06-02

**Authors:** Alexander G. Kuzma-Hunt, Vipra Shah, Sierra DiMarco, Keith A. Russell, Dean H. Betts, Thomas G. Koch

**Affiliations:** ^1^ Department of Biomedical Sciences, Ontario Veterinary College, University of Guelph, Guelph, Canada.; ^2^ Department of Obstetrics and Gynaecology, University of Western Ontario, London, Canada.; ^3^ Department of Physiology and Pharmacology, University of Western Ontario, London, Canada.; ^4^ The Children's Health Research Institute - Lawson Health Research Institute, London, Canada.

**Keywords:** canine, canine induced pluripotent stem cell, cell therapy, reprogramming, stem cell, epigenetic

## Abstract

Induced pluripotent stem cells (iPSCs) are produced by resetting the epigenetic and transcriptional landscapes of somatic cells to express the endogenous pluripotency network and revert them back to an undifferentiated state. The reduced ethical concerns associated with iPSCs and their capacity for extensive self-renewal and differentiation make them an unparalleled resource for drug discovery, disease modeling, and novel therapies. Canines (c) share many human diseases and environmental exposures, making them a superior translational model for drug screening and investigating human pathologies compared to other mammals. However, well-defined protocols for legitimate ciPSC production are lacking. Problems during canine somatic cell reprogramming (SCR) yield putative ciPSCs with incomplete pluripotency, at very low efficiencies. Despite the value of ciPSCs, the molecular mechanisms underlying their unsuccessful production and how these may be addressed have not been fully elucidated. Factors, including cost, safety, and feasibility, may also limit the widespread clinical adoption of ciPSCs for treating canine disease. The purpose of this narrative review is to identify barriers to canine SCR on molecular and cellular levels, using comparative research to inform potential solutions to their use in both research and clinical contexts. Current research is opening new doors for the application of ciPSCs in regenerative medicine for the mutual benefit of veterinary and human medicine.

## Introduction

Dogs are excellent models for studying regenerative medicine in humans [1,2] because: (1) 58% of canine genetic diseases are orthologs of human diseases [3,4]; (2) the life span of dogs permits long-term studies [5]; (3) many genomic, anatomical, and physiological similarities exist between dogs and humans [6]; and (4) numerous diseases potentially treatable with induced pluripotent stem cells (iPSCs) occur in both dogs and humans, including cancers, diabetes, autoimmune diseases, and cardiomyopathies [7–11]. The strong bio-comparability between domesticated dogs and humans is likely a result of exposure to similar environmental factors, permitting better control over such confounders when performing cross-species comparisons [12].

Stem cell research and applications in medicine have greatly evolved over the last decade. One of the most prominent discoveries was made by Takahashi and Yamanaka in 2006, when they reverted mouse embryonic fibroblasts (MEFs) to a pluripotent state by forcing the ectopic expression of four pluripotent transcription factors (TFs), including OCT4, SOX2, KLF4, and c-MYC (OSKM), which inactivated cell-fate genes and reactivated the endogenous pluripotency transcriptional network [13]. The advent of these iPSCs opened new research directions in the field of regenerative medicine by avoiding potential ethical concerns associated with the sourcing of pluripotent cells from preimplantation embryos.

iPSCs resemble embryonic stem cells (ESCs) in that they exhibit indefinite proliferative capacity, express ESC-specific genes, and can potentially differentiate into specialized cell types from all three germ layers [13–15]. Thus, iPSCs may be used to restore damaged tissue by replacing lost/damaged cells directly or directing regeneration indirectly through paracrine release of cytokines and growth factors [16,17]. These functions make iPSCs a valuable candidate treatment for many veterinary and human pathologies that are not adequately treated currently.

Canine (c) iPSCs thus represent a highly translational model for studying human disease through disease modeling and drug screening in vitro. Although several articles describe the generation of ciPSCs [2,5,15,18–35], obtaining “high-quality” *bona fide* ciPSCs remains elusive with groups reporting evidence of incomplete reprogramming. Therefore, it is important to investigate how canine-specific regulators of cellular reprogramming may create barriers to the generation of ciPSCs with the aim of developing protocols that are reproducible and able to address species-specific differences in iPSC generation.

Despite their potential in clinical applications, there are knowledge gaps in evaluating the therapeutic uses of ciPSCs and overcoming practical barriers to their implementation. The purpose of this review is to: (1) provide an overview of current techniques being used to generate ciPSCs; (2) discuss reprogramming of canine somatic cells to ciPSCs and potential solutions for overcoming the molecular mechanisms associated with ineffective reprogramming, and (3) address the clinical utility of ciPSCs and barriers to their application. Evaluating these factors may take us one step closer to reaping the benefits of ciPSCs in veterinary and human regenerative medicine.

## Current Methodologies for ciPSC Production

**FIG. 1.**
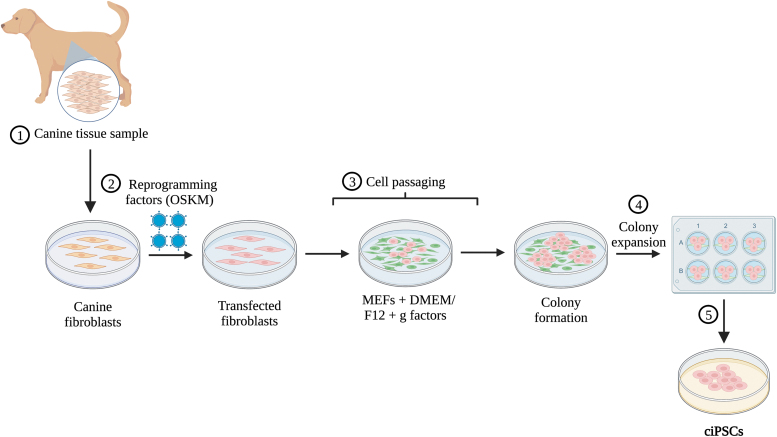
Overview of ciPSC production. (1) A tissue sample is collected from a donor, for example, skin fibroblasts. (2) The sample is transfected with reprogramming vectors generally containing OSKM, using viral or nonviral methods. (3) Cells are passaged onto mitotically inactivated MEF feeder cells, suspended in iPSC media that consists of DMEM/F12 plus any growth factors (LIF and/or bFGF). (4) Once putative colonies begin to form, they are selected to be expanded in six-well plates with MEFs and iPSC media. (5) After induced pluripotent colonies show robust growth, they are ready to be validated and used for either research purposes or clinical applications. ciPSC, canine induced pluripotent stem cell; MEF, mouse embryonic fibroblast; LIF, leukemia inhibitory factor; bFGF, basic fibroblast growth factor; DMEM, Dulbecco's modified Eagle's medium.

**FIG. 2.**
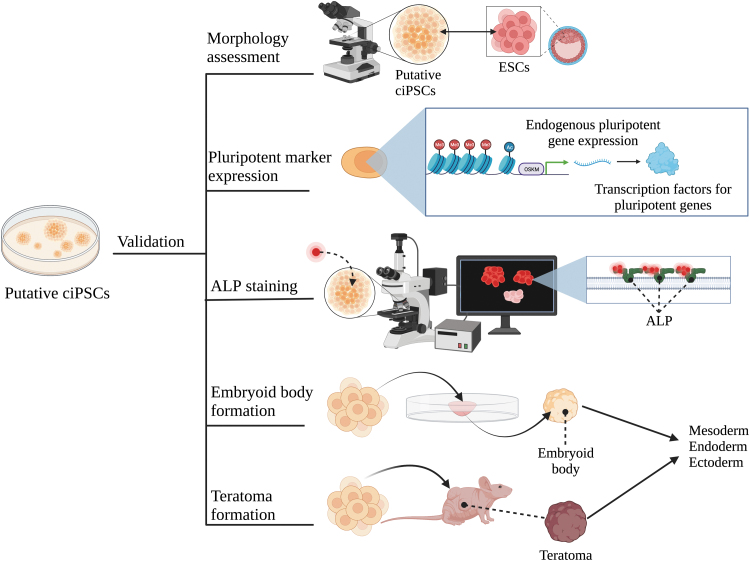
Methodologies for validating pluripotency. Validating the pluripotency status of putative ciPSCs may involve a combination of methods, including morphological assessment, pluripotency marker expression, ALP staining, embryoid body formation, and teratoma formation. ALP, alkaline phosphatase.

To optimize the production of ciPSCs, it is important to assess the efficacy and methodologies of current techniques. Current methods for ciPSC production are primarily derived from the protocol developed originally by Yamanaka et al. (2006) in mice (Fig. 1). Although there is no standard procedure, most methods follow a similar general protocol, as follows: (1) tissue is collected by surgical means and cells are removed for culturing [5,21]; (2) cells are transfected one or more times with vectors containing reprogramming factors [5,21]; (3) post-transfection, cells are transferred onto culture plates with mitotically-inactivated feeder cells such as MEFs or synthetic alternatives in iPSC media, usually consisting of Dulbecco's modified Eagle's medium/Nutrient Mixture F-12 (DMEM/F-12) and growth factors [5,21]; (4) once colonies begin to form, they are expanded in plates with MEFs and iPSC media [5]; (5) colonies that show robust growth are then mechanically passaged for continued expansion [5]; (6) following expansion, the pluripotency status of resultant ESC-like colonies is validated using various techniques (Fig. 2) [36].

### Source of donor cells

ciPSCs have been produced from a wide range of differentiated cells, such as canine adult dermal and testicular fibroblasts (cAFs), adipose mesenchymal stem cells (MSCs), adipose stromal cells, canine embryonic fibroblasts (cEFs), and peripheral blood mononuclear cells (PBMCs) (Table 1) [4,5,15,19,21–25,27,29,35,37–40]. These cells have been collected for the purposes of ciPSC generation from a range of dog breeds, including the Weimaraner, beagle, Jindo, German shorthaired pointer, and poodle [4,5,15,19,21–25,27,35,37–40].

**Table 1.** Summary of the Starting Tissues, Reprogramming Methods, and Validation of Pluripotency Techniques Used for the Production of Canine Induced Pluripotent Stem Cells

Reference	Original starting tissue	Transfection vector	Reprogramming factors	Growth factors	Pluripotency marker expression	In vitro differentiation	Teratoma formation
Yoshimatsu et al. (2021) [48]	Ear skin fibroblasts	Episomal	hOSKM, LIN28, NANOG, KLF2, KDM4D, GLIS1, mp53DD	LIF, bFGF	Yes	EB formation	Yes
Lee et al. (2011) [22]	Adipose stromal cells, Skin fibroblast	Lentivirus	hOSKM	LIF, bFGF	Yes	EB formation	Yes
Whitworth et al. (2012) [5]	Skin fibroblasts	Lentivirus	hOSKM, LIN28, NANOG	LIF, bFGF	Yes	EB formation	No
Luo et al. (2011) [4]	Testicular fibroblasts	Lentivirus	hOSKM	LIF, bFGF	Yes	EB formation	No
Gonçalves et al. (2017) [23]	Embryonic fibroblasts	Lentivirus	hOSKM, mOSKM	bFGF	Yes	EB formation	Yes
Nishimura et al. (2017) [37]	Embryonic fibroblasts	Lentivirus	mOSKM	bFGF	Yes	EB formation	No
Nishimura et al. (2013) [38]	Embryonic fibroblasts	Lentivirus	hOSKM	LIF, bFGF	Yes	EB formation	Not tested
Baird et al. (2015) [21]	Adipose-derived MSCs	Retrovirus	hOSKM	LIF, bFGF	Yes	EB formation	Not tested
Koh et al. (2013) [15]	Skin fibroblasts	Retrovirus	mOSKM	LIF, bFGF	Yes	EB formation	No
Shimada et al. (2010) [35]	Embryonic fibroblasts	Retrovirus	cOSKM	LIF, bFGF, 3i	Yes	EB formation	Not tested
Kwon et al. (2012) [39]	Fetal and adult fibroblasts	Retrovirus	hOSKM	LIF, bFGF	Yes	EB formation	Not tested
Tsukamoto et al. (2018) [19]	Embryonic fibroblasts	Sendai virus	hOSKM	LIF, bFGF	Yes	EB formation	Yes
Kimura et al. (2021) [25]	PBMCs	Sendai virus	hOSKM	LIF, bFGF	Yes	EB formation	Yes
Chow et al. (2017) [27]	Skin fibroblasts	Sendai virus	hOSK	LIF	Yes	EB formation	Yes
Kim et al. (2020) [24]	Skin fibroblasts	VEE RNA virus	hOSK, GLIS1	LIF, bFGF	Yes	Not tested	Not tested

hOSKM, Human OCT4, SOX2, KLF4, c-MYC; LIF, leukemia inhibitory factor; bFGF, basic fibroblast growth factor; EB, embryoid body; mOSKM, murine OCT4, SOX2, KLF4, c-MYC; MSC, mesenchymal stem cell; cOSKM, canine OCT4, SOX2, KLF4, c-MYC; 3i, glycogen synthase kinase-3 beta; PBMC, peripheral blood mononuclear cell; hOSK, human OCT4, SOX2, KLF4; VEE, venezuelan equine encephalitis.

Although there is a variety of tissues that can be used to extract cells for generating ciPSCs, it is unclear if cells from certain tissues produce *bona fide* ciPSCs at higher efficiencies compared to others. However, comparative reports in humans indicate that different somatic cells possess their own advantages and disadvantages for generating hiPSCs [41]. Fibroblasts appear to be the most common cell type used to generate hiPSCs because of their availability from skin biopsy and reliable outcomes following cultivation, propagation, and cryopreservation [41]. Despite their popularity, human fibroblast collection typically involves a skin biopsy, which many consider an invasive procedure. Hair follicle-derived keratinocytes have been proposed to be a less invasive source of cells for generating hiPSCs at 100-fold higher reprogramming efficiencies (∼1%–2%) compared to fibroblasts [41–43]. Although there is a lack of strong evidence comparing ciPSC efficiency rates generated from different somatic cells, most cell types, especially PBMCs and cAFs, are abundant and easy to collect depending on the breed. However, there are no clear links between a dog's breed or sex and its ability to produce viable ciPSC colonies.

Future research may explore differences in the molecular profiles and production efficiencies of ciPSCs produced from different breeds or sexes. Neutering and spaying of canines present minimally invasive opportunities to collect reprogrammable cells to investigate potential sex- and breed-dependent differences in ciPSC production and for banking tissue for future iPSC generation needs [4].

### Reprogramming vectors

Once donor cells are harvested, they are transfected/transduced with vectors containing reprogramming factors required to revert cells to a pluripotent state. Reprogramming vectors can be viral or nonviral, but viral vectors have been utilized in most canine reprogramming cases as summarized in Table 1. To date, vectors used to generate ciPSCs have included unspecified retroviruses [2,15,21,33,35], lentiviruses [4,5,21,23,28,38], Sendai viruses [19,25,27,29,32], Venezuelan equine encephalitis (VEE) virus [24], and episomes [40].

Although retroviruses such as lentiviruses have been the most popular transduction vector, there are concerns associated with transgene integration into the host genome [4,5,15,21–23,35,38,44], which can lead to the re-expression of transgenes in iPSCs and potential tumorigenesis [24,29]. Nonintegrating Sendai viruses may circumvent issues related to tumorigenesis because they are nonintegrating RNA viruses, meaning that transgene expression remains in the cytoplasm [19,25,27]. Therefore, expression of exogenous genes by Sendai viruses is transient and can be endogenously limited by expressing cytoplasmic gene silencer micro-RNAs (miRs), such as miR-302, in pluripotent cells [19,25,27,45]. Sendai viruses are an attractive reprogramming vector because they allow for the creation of footprint-free ciPSCs.

Unfortunately, mixed results have been reported by groups using Sendai viruses for ciPSC production. Chow et al. (2017) first reported their use, but the efficiency of colony generation was very low, forming only one viable colony [27]. Better success rates were reported by Tsukamoto et al. (2018), with a reprogramming efficiency of 0.02%, which is significantly higher than the efficiency using lentivirus vectors reported by Whitworth et al. (2012) of 0.0007% [5,19]. More recently, Nishimura et al. (2017) reported lentiviral reprogramming efficiency rates of 0.048% [37]. However, the highest reported efficiencies for ciPSC colony production are still a lot lower than studies reporting low efficiencies in both human and mouse pluripotency reprogramming [46,47].

VEE virus is another nonintegrating vector that has been used for reprogramming cells to ciPSCs. The VEE replicon is a single-stranded positive sense RNA with a 5′ cap and poly (A) tail and avoids issues associated with integration into genomic DNA because it does not use a DNA intermediate [24]. It is also more cost and time effective to synthesize the VEE replicon than Sendai virus particles [24]. This method has been successfully used to generate human iPSCs and was recently tested in canine cell lines [24]. Despite expressing pluripotency factors, the ciPSCs that were generated using the VEE virus were believed to only be partially reprogrammed because their morphology did not resemble that of cESCs [24]. In addition, the VEE replicon can induce interferon-related innate immune responses and has a very large replicon (11 kb), which may pose barriers to transfection [24]. Although the use of the VEE virus shows promise for ciPSC generation, further experimentation is necessary to assess whether ciPSCs reprogrammed with the VEE virus are truly pluripotent.

More recently, nonviral, integration-free episomal vectors have been used to reprogram canine cells. Episomal vectors replicate extrachromosomally and are diluted out of cells during division [40]. ciPSCs produced by Yoshimatsu et al. (2021) using episomal vectors showed markers of pluripotency and functioned as fully pluripotent ciPSCs [48]. While more studies are needed to validate the use of episomal vectors, this is a new promising avenue for potential ciPSC generation.

### Reprogramming factors

While the most widely used set of reprogramming factors are OSKM [4,15,19,21–23,25,27,35,37–39], some studies have used alternative or additional reprogramming factors (Table 1), with LIN28 and NANOG being the most common [5]. Yoshimatsu et al. (2021) combined 10 factors to create their ciPSC line, including OSKM, LIN28, mp53DD, KLF2, NANOG, KDM4D, and GLIS1 [40]. In other studies, such as Kim et al. (2020) and Chow et al. (2018), c-MYC was removed from the OSKM cocktail due to its tumorigenic potential. Although human and mouse reprogramming factors are most used to produce ciPSCs (Table 1), Shimada et al. (2010) successfully used reprogramming factors of canine origin [35]. However, it remains unclear if mouse- or human-derived reprogramming factors are best suited for ciPSC derivation.

### Validation of pluripotency status in ciPSC colonies

Validating the pluripotency of putative iPSC colonies involves identifying overlapping characteristics with ESCs. Once colonies have formed, there are several ways to confirm that ciPSCs are indeed pluripotent, including morphological assessment, pluripotency marker expression, alkaline phosphatase (ALP) staining, embryoid body formation, and teratoma formation (Fig. 2) [36].

The majority of studies that produced ciPSCs considered in this review showed that the morphology of ciPSCs was similar to that of hESCs with well-defined borders, cobblestone appearance, and high nuclear-to-cytoplasmic ratio [49], as well as expression of endogenous pluripotency markers *OCT4*, *SOX2*, *NANOG*, *TRA-1-60*, *TRA-1-81*, *SSEA-1*, and *SSEA-4*, (Table 1) [4,5,15,19,21–25,27,35,37–40]. Staining cells for ALP, which is a membrane bound glycoprotein highly expressed in ESCs, is another commonly used method for validating pluripotency and is used by many studies within this review [4,5,15,19,21–25,27,35,37–40,50]. To test the differentiation potency of ciPSCs in vitro, most groups formed embryoid bodies, which are three dimensional aggregates of pluripotent stem cells that give rise to progenitors in all three germ layers – mesoderm, ectoderm, and endoderm [4,5,15,19,21–25,27,35,37–40].

A subset of studies also tested for teratoma formation, which can be used to test their in vivo differentiation potential, by generating nonmalignant tumors consisting of a disorganized mixture of cells and small foci of tissue composed of cells from all three embryonic germ layers [19,22,23,25,27,48,51]. Most studies in this review were successful in forming complete teratomas [19,22,23,25,27,48]. However, Koh et al. (2013) formed solid tumors that had derivatives of all three germ layers but failed to reach complex epithelial structures [15]. In contrast, Whitworth et al. (2012) found that partially reprogrammed cells were unable to form teratomas, but cells with high expression of transgenes formed neoplasms that resembled germ cell tumors [5]. In addition, the true meaning of these trilineage differentiation assays may be questioned considering that most groups report continued transgene expression or only partial transgene silencing in ciPSCs [4,15,19,23,24,47]. Few groups have successfully achieved complete transgene silencing in ciPSCs [5,21,25,38] indicating that most reported cell lines are not fully reprogrammed.

Inefficient or incomplete canine somatic cell reprogramming (SCR) could be attributed to a multitude of factors related to either cellular identity such as chromatin accessibility and changes to the epigenome or methodology such as the use of certain media components and reprogramming factors. Investigating molecular mechanisms capable of interfering with canine cell reprogramming and identifying species–species differences in iPSC production may provide a strong basis for overcoming barriers in ciPSC production.

## Addressing Current Limitations in Reprogramming Canine Cells to ciPSCs

### Molecular mechanisms underlying SCR of canine cells

**FIG. 3.**
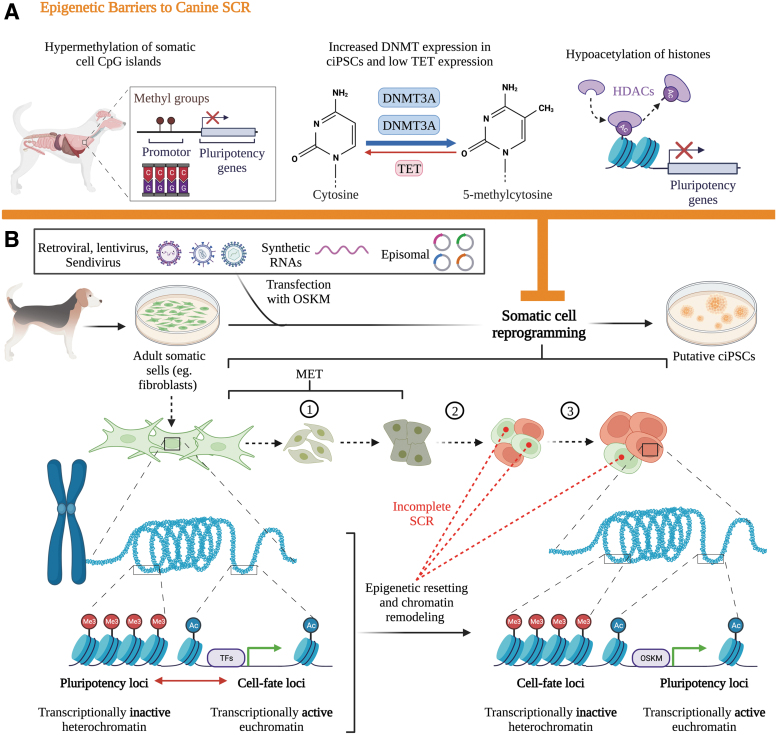
A schematic representation of canine epigenetic barriers **(A)** preventing somatic cell reprogramming **(B)** The unique epigenetic landscape of canine somatic cells, including hypermethylation of CpG islands in most tissues, increased expression of DMNTs, and hypoacetylation of histones all impede successful reprogramming to pluripotency. SCR occurs in stages, including (1) initiation, largely defined by the MET; (2) maturation; and (3) stabilization of pluripotency. Chromatin-state remodeling involves “closing” somatic fate loci and “opening” pluripotency loci. Incomplete canine SCR may be a result of failed epigenetic resetting and chromatin remodeling. MET, mesenchymal to epithelial transition; SCR, somatic cell reprogramming.

SCR is governed by a series of molecular events that rapidly inactivate somatic-fate loci responsible for cellular differentiation and progressively activate pluripotency loci (Fig. 3) [52,53]. Global changes to chromatin accessibility, resulting from epigenetic changes to these loci, are a key determinant of successful cellular reprogramming [2,28,32,53,54] (Fig. 3). Specifically, the chromatin of adult somatic cells is remodeled by expressing epigenetic repressive markers on lineage-specific genes while removing such markers on pluripotency genes [52–56].

Chromatin remodeling can be conceptualized as a transition from an “open to closed” (OC) state near cell-fate genes and a “closed to open” (CO) state near pluripotency genes [53] (Fig. 3B). Epigenetic mechanisms, such as DNA methylation and histone post-translational modifications (PTMs), may work both synergistically and independently to reorganize somatic cell chromatin [52,55–57]. These changes involve initiation, maturation, and stabilization of cells as they acquire pluripotency [32,54] (Fig. 3B). However, the same epigenetic mechanisms are also responsible for maintaining cell identity (Fig. 3A). Therefore, species-specific differences in the epigenetic landscape responsible for maintaining cellular identity may underlie the difficulties in canine SCR compared to rodents and humans.

In contrast to changes occurring during SCR in mice [54], alterations to the epigenome during ciPSC generation are yet to be well characterized. Recent studies have investigated chromatin dynamics during ciPSC generation [2] and the effects of manipulating various epigenetic regulators such as histone deacetylases (HDACs) [28] and ten-eleven translocation (TET) enzymes [32] that modulate chromatin accessibility by changing the degree of histone acetylation and DNA methylation, respectively. Other studies have investigated various combinations of small molecule compounds (SMCs) known to influence epigenetic regulators [24,25,29,32,48,58]. Defining the pluripotent state of ciPSCs may also provide insight into canine-specific regulators of SCR, considering that there are distinct epigenetic differences between primed and naive states of pluripotency seen in postimplantation epiblast cells and the preimplantation inner cell mass, respectively [33,59,60].

### Chromatin accessibility and epigenetic barriers to cellular reprogramming

The epigenome consists of molecular regulators that modify DNA and nucleosome-forming histones, which influence gene expression without changing the DNA sequence [57]. Epigenetic mechanisms play a critical role in the reorganization of chromatin during cellular reprogramming by either enhancing or repressing genes required for pluripotency induction, cellular differentiation, and development. Epigenetic mechanisms recently studied in the context of ciPSC production include histone PTMs [28] and DNA methylation [32].

DNA methylation and histone PTMs, namely histone methylation and acetylation, determine whether chromatin assumes a compact, transcriptionally inactive heterochromatin state or a loose, transcriptionally active euchromatin state [57] (Fig. 3B). Heterochromatin-associated modifications include methylation of cytosine to 5-methylcytosine (5-mC) through DNA-methyltransferases and deacetylation of lysine residues on histones by HDACs, which inhibit TFs from binding DNA [57].

The activity of promoters for pluripotency genes is also determined by the degree of methylation within CpG islands that safeguard cell identity along with DNA methylation and hypoacetylation, contributing to the unique epigenetic landscape of canine somatic cells [32]. In dogs, CpG islands are highly methylated relative to CpG islands in other species [61]. In fact, unpublished data from Tobias found that a subset of reprogramming associated orthologs was more CpG rich in the canine genome relative to other species such as mouse and human (IC Tobias, thesis dissertation). Incomplete demethylation of CpG rich regions, including the *NANOG* promoter, was thought to be responsible for generating pseudo-reprogrammed canine cells that were still expressing pluripotency transgenes [32].

Compared to iPSCs from humans and mice, DNMT3A is upregulated in ciPSCs [33], and hypoacetylation of histones impairs chromatin accessibility [28] (Fig. 3A). Questa et al. (2020) suggested that these factors impede effective reprogramming of canine cells by causing key pluripotency genes to remain inactive while maintaining the activity of cell-fate genes [2]. Therefore, manipulating DNA methylation and histone acetylation during canine SCR, while identifying candidate genes that fail to “close” or “open”, may reveal their importance in generating ciPSCs.

### Manipulating histone acetylation and DNA methylation during ciPSC generation

Moshref et al. (2021) investigated the effects of manipulating histone acetylation during cEF reprogramming *via* lentiviral transduction of OSKM to generate ciPSCs [28]. Panobinostat (LBH589) and vitamin C/ascorbic acid (AA) were used to inhibit HDACs in the reprogramming media. LBH589 (1 nM) significantly reduced HDAC activity and increased the acetylation of histones H3K27 and H3K9, which influence enhancer and promoter activation during reprogramming, respectively [62,63]. LBH589 + AA treatments caused a consistent increase in chromatin accessibility in treated cEFs relative to controls, suggesting that genes associated with cell fate, such as *TGFB1* and *BMP4*, were more closed in the treatment group. In contrast, TF-binding motifs were opened for both cell-identity and reprogramming factors, such as AP1 and CEBP. However, the same treatment did not change the reprogramming efficiency of cEFs.

Taken together, the results of Moshref et al. (2021) indicate that treatment with LBH589 + AA can reliably increase chromatin accessibility during cEF reprogramming to ciPSCs and, importantly, that manipulating individual epigenetic modulators such as HDACs may not be enough to improve the reprogramming efficiency of cEFs [28]. The cooperativity of histone PTMs and DNA methylation in regulating chromatin accessibility could explain, in part, the lack of changes in reprogramming efficiency.

Although Moshref et al. (2021) observed changes in chromatin accessibility in cEFs, this may not be the case for cAFs, since canine cells derived from adult and embryonic tissues differ significantly in their chromatin accessibility and ability to reprogram to ciPSCs [2,29,30,32]. Therefore, the absence of a cAF control group in Moshref et al. (2021) limits the generalizability of their results, given that adult cells are more resistant to reprogramming compared to cells of embryonic tissues. Prior reports showed that the use of similar HDAC inhibitors as Moshref et al. (2021) improved SCR efficiency in humans and mice [64–67], suggesting the need for canine-specific iPSC protocols.

Interestingly, Moshref et al. (2021) found that AP1 and AP-2 TF binding motifs were enriched in cEFs following LBH589 + AA treatment. This is of relevance because AP1 related TFs inhibit SCR [53], whereas AP-2 TF binding motifs in the *OCT4* enhancer region are enriched in naive pluripotent cells [68]. Consistent with findings from Moshref et al. (2021), the same researchers previously observed an increase in enrichment for AP1 c-Jun in cEFs and ciPSCs relative to adult stromal cells [2]. Furthermore, Markov et al. (2021) found that AP1 c-Jun impeded the reprogramming of human fibroblasts to iPSCs by increasing the accessibility of enhancers for fibroblast-specific gene expression and inhibiting OCT4 as part of a protein complex with MBD3 [69]. Markov et al. (2021) also demonstrated that vectors expressing a dominant, inducible AP1 mutant (dnAP1) in addition to SOX2 and KFL4 were viable substitutes for OCT4 and improved reprogramming by reducing the accessibility and expression of fibroblast-specific genes [69].

Although species differences likely exist, it is possible that AP1 enrichment in both ciPSCs and cEFs [2,28] may have a similar somatic-gatekeeper function as it does in humans [69], making its modification a potential means of improving canine cellular reprogramming.

Tobias et al. (2020) characterized the dependence of critical steps in the early stages of canine SCR on modulators of DNA methylation [32]. They investigated cEFs, cAFs, canine partially reprogrammed cells (cPRs), and cESCs. A Sendai virus expressing OSKM was used to transduce cEFs and cAFs, while methylation was manipulated by the addition of 100 μM AA and 0.1 nM retinoic acid (RA) to the reprogramming media. RA and AA were chosen for their capacity to regulate the catalytic activity and abundance of TET enzymes, which demethylate pluripotent genes, using 2-oxoglutarate (2-OG) during iPSC generation [70]. Specifically, TET enzymes oxidize 5-mC to 5-hydroxymethylcytosine (5-hmC), meaning that the ratio of 5-mC to 5-hmC can be used as an indicator of TET activity and demethylation [71].

Tobias et al. (2020) found that cPRs derived from cAFs had reduced pluripotent gene expression, increased methylation at the *NANOG* promoter, and a lower level of global 5-hmC compared to cESCs. In addition, cEFs had higher levels of 5-hmC and greater expression levels of TETs 1, 2, and 3 compared with cAFs. Both results suggest that regulators of DNA methylation may prevent successful canine SCR. The researchers also demonstrated that initiation of cEF reprogramming, largely defined by the mesenchymal-to-epithelial transition (Fig. 3B), can be bolstered by AA + RA treatment, which increased 5-hmC accumulation in canine fibroblasts and initial pluripotent stem cell colony formation. These findings contrast with those of Moshref et al. (2021) regarding the utility of AA in improving the efficiency of ciPSC generation, perhaps because Moshref et al. (2021) used a concentration of 150 μM AA, which is above the cytotoxic limit (100 μM), whereas Tobias et al. (2020) used 100 μM.

Tobias et al. (2020) also identified stage-dependent positive regulators of canine SCR, including *TGFB2*, *SMAD2*, *KFL4*, *NR0B1*, and *CDH1* associated with initiation and *TGFB1*, *LIN28A*, *EZH2*, and *EED* associated with maturation. These factors were depleted in cPRs relative to cESCs making them strong candidates for ectopic coexpression with OSKM in ciPSC generation. In addition, DPPA5, which was one of the pluripotency factors distinguishing cESCs from reprogramming primary colonies, is among proteins that play an important role in cellular reprogramming [32,72]. Specifically, coexpression of *DPPA2* and *DPPA4* with OSKM has yielded reprogramming efficiencies that exceed 80% in mice and humans [72] warranting further investigation into DPPA proteins during ciPSC generation.

More recently, Yamazaki et al. (2021) characterized the methylome of 16 canine somatic tissues, which indicated that dogs possess a higher degree of CpG island methylation relative to other species [61]. Of the 130,861 CpG sites analyzed, over 70% were highly methylated (52.4%–64.6% of CpG islets), while less than 30% had the lower levels (22.5%–28% of CpG islets) seen in other species [61]. Thus, future research on improving ciPSC generation could involve targeting TET-regulated genes [32] and selecting specific donor tissues with low levels of CpG methylation such as spleen [61]. However, DNA methylation status of the tissues/cells will change with the age of the donor and must be considered in the selection process [61].

### Chromatin-state dynamics: “Opening” pluripotent genes and “Closing” differentiation genes

Having identified epigenetic modulators that likely impede successful canine cellular reprogramming, it is important to determine the genes affected and their role in pluripotency. Questa et al. (2021) defined changes in global chromatin accessibility during canine SCR [2]. Following transduction of canine dermal fibroblasts (cDFs), cEFs, and adult stromal cells (cASCs) with retroviral vectors expressing OSKM, it was found that cEFs, but not cASCs or cDFs, reprogrammed to ciPSCs due to differences in chromatin accessibility. Questa et al. (2021) identified differentially accessible TF motifs, as well as OC and CO genes between cEFs and ciPSCs. By making a similar comparison between cDFs and cEFs, they were able to identify which of the genes and TF-motifs either failed to open (FO) or failed to close (FC), representing candidates responsible for impeding SCR. They were able to identify several TF binding motifs and 76 FO or FC candidate genes (Table 2). Their results indicate that FC genes made up most candidate genes, suggesting that the accessibility of cell-fate determining genes is the main barrier to canine SCR.

**Table 2.** Candidate Reprogramming Barrier Genes

Candidate fail to close genes			Candidate fail to open genes
*ADGRE1*	*GUSB*	*PRKDC*	*ACAN*
*ALCAM*	*HIPK2*	*PSMB7*	*ADGRL2*
*ANGPT1*	*HMGA1*	*QKI*	*AVPR1A*
*AOXA*	*HRAS*	*RAB10*	*B3GAT2*
*BCAN*	*IL2RA*	*RAB22A*	*BCL2*
*BCL2L1*	*JUP*	*RBM12*	*CTSC*
*BMPR1B*	*KCNMB1*	*RECK*	*DCT*
*CAV1*	*KDR*	*RETREG1*	*DSG3*
*CCL17*	*LEPR*	*SERPINE1*	*GMPS*
*COMMD1*	*MAPK1*	*SERPINH1*	*GNAQ*
*CPT1A*	*MAPT*	*SLC6A2*	*LIPC*
*CRYBA1*	*MMP11*	*SMAD3*	*NOX3*
*DAG1*	*NDRG1*	*SREBF1*	*PNPLA1*
*DDX39B*	*NFKB1*	*STK38L*	*PSEN1*
*DKK3*	*NUDT3*	*SULF1*	*SIRT5*
*DPT*	*PDX1*	*UBE2N*	*SLC7A1*
*EIF3L*	*PLCE1*	*UNK*	*SPINK5*
*FBN1*	*PPARG*	*VEGFA*	*TACR1*
*GHR*	*PPP3CA*	*XYLT1*	*TACR3*

In contrast to genes that “fail to open” (FO), “fail to close” (FC) genes make up the majority of candidates impeding canine SCR. From Questa et al. (2021). Licensed under CC by 4.0. No changes were made. To view a copy of this license, visit http://creativecommons.org/licenses/by/4.0/

The results of Questa et al. (2021) are supported by previous studies in mice suggesting that OC genes are related to somatic cell fate, while CO genes are enriched for OSKM motifs [53]. FO or FC genes in canine SCR could be targeted by gene editing technologies such as CRISPR-Cas9. Although this technology has not been used in the generation of ciPSCs, it has been successful in mice. Black et al. (2016) and Liu et al. (2018) utilized CRISPR-Cas9 to manipulate epigenetic loci, converting fibroblasts to neuronal progenitors and remodeling the endogenous *OCT4* and *SOX2* loci to promote cellular reprogramming [73,74]. More recently, Jiang et al. (2022) used a multiplexed CRISPR-Cas9 system to selectively open chromatin loci for silenced genes, including *Gata4*, *Nkx2.5*, and *Tbx5*, facilitating the reprogramming of mouse fibroblasts to cardiac progenitor cells [75]. This system bypassed pluripotency while retaining the proliferative nature of cardiac progenitor cells—another potentially viable solution for overcoming problems with canine SCR [75].

Future studies could investigate how a CRISPR-Cas9 system, multiplexed for pluripotency genes such as OSKM, or the candidate genes proposed by Questa et al. (2021) (Table 2), could be used to overcome epigenetic barriers to canine SCR.

## Effective Canine SCR Protocols and Defining the Final Pluripotent State of ciPSCs

### Addressing low efficiencies and the need for standardized protocols for canine SCR

Variability between protocols for canine SCR makes obtaining consistent ciPSCs difficult, resulting in functional differences between cells, as seen in human iPSCs [76]. The main source of variability appears to be the choice of reprogramming media and the vectors used to transduce cells.

Media choice significantly affects canine SCR during somatic cell nuclear transfer [58] and impacts the efficiency of ciPSC production [25,29,30]. Various media and SMC cocktails have been used for canine SCR, such as knockout serum replacement (KSR) media with bFGF ± LIF [15,19,21,23,24,27–29,35] and neural basal (N2B27) media [18,25,30,48]. Based on reprogramming efficiency rates, N2B27-based media in addition to SMCs (10 mM Y-27632, 0.5 mM PD0325901, 3 mM CHIR99021, 0.5 mM A-83-01, 10 mM, Forskolin, & 50 mg/mL AA) may be best for reprogramming PBMCs [25] and cAFs [18,30]. However, the inability to control for differences across studies, such as selection of transfection vectors, SMCs, and the type or age of donor cells, makes accurate comparisons between canine SCR efficiencies extremely difficult. A more productive approach to optimizing the reprogramming media may be to identify similarities between studies with high efficiency rates, an example being the use of forskolin (a cAMP activator [77]), by both Yoshimatsu et al. (2021) and Kimura et al. (2021), despite both studies having very different protocols [25,30].

Studies also vary in the choice of transfection vectors, including lentivirus [4,5,21–23,28,38], unspecified retroviruses [2,15,21,33,35], Sendai virus [19,25,27,29,32], episomal [30], and VEE RNA virus [24] (Table 1). Each technique has strengths and weaknesses, but future comparative research could consider transfection and canine SCR efficiency in a cell-dependent manner to determine the best protocol.

The choice of reprogramming factors is vital, given that variants of OSKM or cotransfection with other pluripotency factors have improved SCR in canine and other species. Yoshimatsu et al. (2021) overcame problems with canine cAF reprogramming by cotransfection with episomal vectors containing GLIS1 and KDM4D, which are associated with higher reprogramming efficiencies in fibroblasts [30]. Interestingly, GLIS1 induces pluripotency through multilevel epigenetic and metabolic remodeling, making it a strong alternative reprogramming factor for c-MYC that may be able to address some of the epigenetic barriers observed in canine SCR.

The metabolomic and epigenetic mechanisms of GLIS1 were discovered by Li et al. (2021) who found that it directly opens chromatin near glycolytic genes and closes the chromatic of somatic genes during SCR of mouse fibroblasts [78]. These GLIS1-induced changes in chromatin structure increase cellular acetyl-CoA and lactate levels because of increased glycolytic flux [78]. Elevated acetyl-CoA and lactate enhances histone acetylation (H3K27Ac) and lactylation (H3K18la) near pluripotency loci, causing them to transition from CO [78]. The metabolic and epigenetic crossover of GLIS1 contrasts the typical OSKM-mediated SCR, which is driven by a “first wave” of TFs from c-MYC and KLF4 followed by a “second wave” from OCT4, SOX2, and KLF4. As described by Li et al. (2021), GLIS1 driven SCR involves a cascade of both endogenous and exogenous factors, involving an epigenome-metabolome-epigenome coordination of histone acetylation and acetylation of “second wave” genes and opening of pluripotent loci [78].

As an alternative to GLIS1, the classic OSKM factors can be mutated to facilitate stronger interactions with DNA, as shown by Borisova et al. (2022) in which a KLF4 L507A variant increased efficiency of mouse embryonic fibroblast reprogramming as a result of a smaller amino acid residue in position 507 [79]. Applying similar strategies to canine SCR may circumvent epigenetic blocks, such as the hypermethylation of pluripotent genes.

### Understanding LIF and FGF dependency

A key aspect of iPSC culture media is the addition of growth factors such as LIF and bFGF. While the survival of mouse and human ESCs is dependent on LIF and bFGF, respectively, ciPSCs seem to require both for the maintenance of pluripotency and cell survival [4,34]. Although most studies use both LIF and bFGF in the culture media when generating ciPSCs (Table 1) [4,5,15,19,21,22,24,25,27,35,38–40], there is conflicting evidence regarding their necessity for use in conjunction or alone. Gonçalves et al. (2017) and Nishimura et al. (2017) generated ciPSCs that were only bFGF dependent [23,37], whereas Whitworth et al. (2012) reported LIF dependence only, indicating that when ciPSCs were grown in the absence of both LIF and bFGF, or with just bFGF, cells spontaneously differentiated into fibroblast-like cells [5]. Surprisingly, when ciPSCs were cultured in both LIF and bFGF, they also differentiated into fibroblast-like cells or underwent cell death [5]. Pluripotency of the colonies was maintained only when cultured with LIF alone [5].

To determine the need for LIF and bFGF by ciPSCs, it is necessary to have a strong understanding of their mechanistic pathways involved in pluripotency [4,34]. LIF supports pluripotency through the activation of the Janus kinase (JAK)/signal transducer and activator of transcription 3 (STAT3) pathway [4,34]. The JAK/STAT pathway is an important signaling pathway for the regulation of germline and somatic stem cell character, while also involved paradoxically in cell growth and cellular differentiation [80,81]. In contrast, bFGF stimulates MEFs to synthesize activin A, which in turn activates the SMAD2/3 pathway, promoting *NANOG* expression [4,34]. By suppressing differentiation factors, NANOG aids in the maintenance of pluripotency while activin A activates the ERK pathway, promoting cell proliferation [4,34].

Human ESCs and mouse epiblast stem cells (EpiSCs) have been shown to require only bFGF [4,5,34]. However, Luo et al. (2011) only saw ciPSC colony formation when both LIF and bFGF were present in the culture media [4]. ciPSC dependence on these two factors was tested using three culture conditions: LIF+/bFGF−, LIF−/bFGF+, and LIF+/bFGF+. When LIF was removed (LIF−/bFGF+), the number of apoptotic cells increased significantly. Pluripotency maintenance was measured by observing the expression levels of *NANOG*, and removal of either LIF or bFGF was sufficient to eliminate the expression of this pluripotency marker. In addition, LIF+/bFGF+ displayed the highest proliferation rates compared to the other culture conditions [4]. These results indicate that both LIF and bFGF are necessary to maintain pluripotency and self-renewal of ciPSCs.

In contrast to Luo et al. (2011), Luo et al. (2016), the same researchers reported that LIF removal, or treatment with JAK inhibitors, did not result in a reduction in pluripotency gene expression or an increase in differentiation genes within ciPSC colonies [34]. Although the direct inhibition of STAT3 decreased the expression of *OCT4*, this still did not increase the expression of differentiation genes. Even though Lou et al. (2016) removed LIF from the culture media, they still used feeder cells that produce LIF, making it difficult to assess the necessity of LIF for ciPSC maintenance. However, Kimura et al. (2021) reported that ciPSCs maintained under feeder-free conditions using StemFit + bFGF but not LIF had equivalent characteristics to ciPSCs maintained using StemFit in the presence of LIF [18]. These results suggest that LIF was not essential for ciPSC maintenance when using StemFit on the feeder-free substrate iMatrix-511 [18].

Taken together, results from Lou et al. (2016) and Kimura et al. (2021) support that, in contrast to mESCs, the activation of STAT3 by LIF in ciPSCs differs in importance when it comes to maintaining pluripotency and that culture conditions necessary for sustaining ciPSCs appear to be more comparable to hESCs than mESCs. However, the results of Luo et al. (2016) still supported their previous study [4] in that ciPSCs underwent apoptosis in the absence of LIF or when the JAK-STAT3 pathway was inhibited.

It remains unclear why some ciPSC colonies need both LIF and bFGF while others do not. Importantly, the dependency on LIF and bFGF seems unrelated to the transfection vector used in the cells or the starting tissue type. However, given that the state of pluripotency seems to dictate, at least in part, LIF and bFGF dependence in human and mouse ESCs, classifying the pluripotent state of ciPSCs may uncover this relationship in dogs.

### Implications of defining the final pluripotent state of ciPSCs

**FIG. 4.**
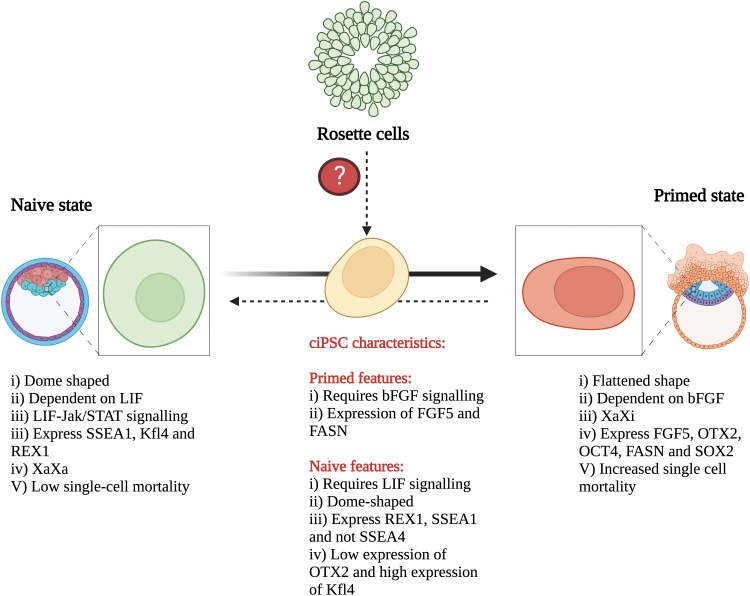
ciPSCs pose characteristics of both naive and primed states of pluripotency. ciPSCs possess similar characteristics to rosette cells, which are a distinct transitory state between naive and primed in mice.

Defining the final pluripotent state of ciPSCs may aid in determining whether species-specific reprogramming factors will improve canine SCR and lead to a better understanding of growth factor requirements. Problems during canine SCR, such as late passage transgene expression, have been suggested to be related, in part, to transduction with mice OSKM [82]. Goncalves et al. (2017) showed that in contrast to mice, using human OSKM resulted in full silencing of the transgene [23], which could be explained by greater similarities between dog and human SCR [83]. Determining if mouse- or human-based OSKM results in better silencing of transgenes is difficult, given the multitude of factors influencing the complete acquisition of pluripotency. However, research to date has been inconclusive concerning the final state of cESCs and ciPSCs. Both primed [4,5,21,22,25,37,38] and naive [15,19,24,28,84,85] state characteristics have been observed, with some studies indicating features of both states [28,32,33,86] (Fig. 4).

Although both primed and naive-state cells can differentiate into any cell type, differences between them include gene expression signatures, chromatin structure, and reliance on exogenous signals [59,87–89]. Naive and primed iPSCs also differ epigenetically, which may explain why naive cells can transition to primed cells a lot easier than the converse [60]. Making this distinction in ciPSCs is important because promoters and enhancers for pluripotency factors such as OCT4 are regulated in a state-dependent manner [60]. For example, Questa et al. (2020) found that the *OCT4* locus in cEF-derived ciPSCs was controlled by its proximal enhancer, indicative of a primed state, rather than its distal enhancer, which is seen in naive cells [2]. Knowledge of which enhancer is being used to regulate *OCT4* expression in ciPSCs may allow for more precise targeting of this key pluripotency gene by TFs such as TFAP2C, which is an AP2 family member known to maintain naive pluripotency in hiPSCs [68] and has been found to be enriched in ciPSCs [28].

Other distinguishing criteria between primed and naive-state cells such as morphology, exogenous signaling dependence, X-chromosome activation, apoptosis, and differentially expressed genes are depicted in Fig. 4. Therefore, determining the pluripotency state of ciPSCs by comparing them with well-defined iPSCs from mice (m) and human (h) may inform superior SCR reprogramming protocols. This was done by Menon et al. (2021) who characterized the pluripotency of ciPSCs derived from cDFs relative to miPSCs and hiPSCs [33]. ciPSCs in LIF, bFGF, and bFGF + LIF exhibited features of both naive and primed states. Naive characteristics included dome-shaped morphology, expression of *REX1*, *SSEA1* but not *SSEA4*, and low expression of *OTX2* and high *KLF4*, whereas primed state indicators included enhanced expression of pluripotency genes in bFGF, increased expression of *FGF5* and *FASN*, and enriched blue fluorescence and bodipy staining (Fig. 4). Menon et al. (2021) concluded that ciPSCs must have been in an intermediate state that was neither naive nor primed since they exhibit properties distinct from miPSCs and hiPSCs [33].

The intermediate state of pluripotency proposed by Menon et al. (2021) could be attributed to unknown species-specific pluripotency factors for dogs, as described for other species [90,91]. Alternatively, the “rosette” state of pluripotency in mice discovered by Neagu et al. (2020) may provide a potential explanation for the intermediate state of ciPSCs [59,92]. The “rosette” state is an intermediate state between naive and primed that is distinct from formative pluripotency, in which cells arrange into a rosette structure and possess apical-basal polarity (Fig. 4). Cells in this state are characterized by the coexpression of both naive-associated *KLF4* and primed-associated *OTX4*, in addition to *SOX2* and *OCT4*, as observed in ciPSCs by Menon et al. (2021) [33,92], minus the presence of apical-basal polarity. Neagu et al. (2020) observed this in mice both in vivo and in vitro, but whether this distinct transitory state exists for other animals, including canine, remains unknown and requires further investigation.

To consistently produce ciPSCs that meet the criteria for pluripotency and safely serve as a clinical tool for translational and veterinary research, the molecular barriers to their production and maintenance need to be addressed. Optimizing SCR will be of the greatest value given the high availability of cells for harvest through minimally invasive techniques. On a molecular level, it appears that canine somatic cells have a unique epigenetic landscape that primarily inhibits the closing of cell fate loci during SCR. In terms of efficiency, there is no standard protocol for ciPSC production, limiting the reliability of findings related to optimizing this process.

It is also important to consider practical limitations of ciPSCs if they are to eventually be used in clinical settings. Important practical considerations include clinical applicability such as the use of ciPSCs for tissue and organ regeneration or immune modulation, the source of cells, safety concerns related to tumorigenesis or immune rejection, and accessibility. A holistic understanding of how ciPSCs could be used clinically, focused on overcoming potential barriers, may better mobilize current research into improving the treatment of canine disease.

## Potential Clinical Utility of ciPSCs

**FIG. 5.**
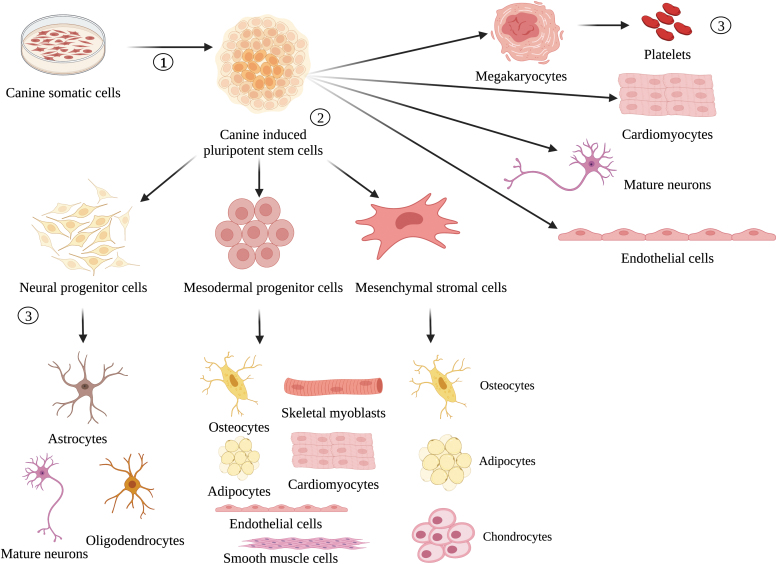
Schematic representation of canine induced pluripotent stem cell differentiation into multiple cell lineages. (1) Somatic cells can be reprogrammed to create ciPSCs. (2) ciPSCs are exposed to lineage- and cell-specific induction media. (3) Differentiated cells are the products of proposed ciPSC therapies.

As displayed in Fig. 5, differentiating ciPSCs into specific cell types is the basis of ciPSC therapy. However, despite the promise of ciPSCs for treating canine disease, their clinical use has not been extensively studied. Lee et al. (2011) completed the first successful autologous transplant of ciPSCs into heart tissue using noninvasive multimodality imaging techniques to monitor cardiac delivery and localization. While this canine model demonstrated successful delivery, undifferentiated ciPSCs are not directly amenable for clinical transplantation due to higher risk of teratoma formation [22]. ciPSCs can be differentiated into either progenitor cells or adult cells such as neural, cardiac, endothelial, and platelets [16,22,38,93–96]. A summary of research studying the clinical applications of ciPSCs to date is shown in Table 3.

**Table 3.** Publications Related to Canine Induced Pluripotent Stem Cell Applications and Cell Type Differentiation

Reference	Reprogramming vector	ciPSC culture conditions	Growth factors	Differentiation media	First differentiated cell type	Second differentiated cell type (if applicable)	Disease of interest (if specified)
Chow et al. (2020) [95]	Sendai virus	DMEM/F12, FBS	bFGF/EGF	Neural Induction Media: DMEM/F12, Antibiotic–Antimycotic, B27, bFGF, EGF, Heparin	Neural progenitor cells	Mature neurons, oligodendrocytes, astrocytes	Spinal cord injury
Chandrasekaran et al. (2021) [94]	Lentivirus	KO DMEM/ F12, KSR	LIF/bFGF/ EGF/Noggin	Neural Induction Media: Neurobasal medium, l-glutamine, B27, N-2, NEAAs, Penicillin-Streptomycin	Neural progenitor cells	Mature neurons	Spinal cord injury
Lee et al. (2011) [22]	Lentivirus	DMEM/F12, KSR	bFGF/LIF	Endothelial Cell Growth Basal Medium-2	Endothelial cells		Myocardial infarction Hindlimb ischemia
Mondal et al. (2022) [96]	Lentivirus	DMEM/F12		Cardiac Induction Media: IMDM/FBS, β mercaptoethanol, azacytidine	Cardiomyocytes		
				Neural Induction Media: DMEM/F12, FBS, forskolin, isobutyl methyl xanthin	Neural Cells		
Quattrocelli et al. (2015) [16]	Retrovirus	KO DMEM/F12	bFGF	MiP Induction: IMEM/FBS, Penicillin Streptomycin, l-glutamine, thioglycerol, ascorbic acid, holo-transferrin	Mesodermal progenitor cells		Muscular dystrophies
				Cardiac Induction: coculture w/neonatal cardiomyocytes in DMEM, FBS, Penicillin-Streptomycin, l-glutamine		Cardiomyocytes	
				Skeletal myogenic Induction: coculture with fetal myoblasts in RPMI, DMEM		Skeletal myoblasts	
				Osteogenesis Differentiation Kit		Osteocytes	
				Adipogenesis Differentiation Kit		Adipocytes	
				Smooth Muscle Induction Media: DMEM, TGF-β1		Smooth muscle cells	
				Endothelial Cell Growth Basal Medium-2		Endothelial cells	
Nishimura et al. (2013) [38]	Lentivirus	DMEM/F12, KSR	bFGF/LIF	Embryonic stem cell differentiation medium with VEGF	Mature megakaryocytes	Functional platelets	Thrombocytopenia
Chow et al. (2017) [27]	Sendai virus	KO DMEM, FBS	LIF	MSC Medium: DMEM/FBS, SB 431542	Mesenchymal stromal cells		Inflammatory disorders
				Adipogenesis Differentiation Kit		Adipocytes	
				Chondrogenesis Differentiation Kit		Chondrocytes	
				Osteogenesis Differentiation Kit		Osteocytes	
Shahsavari et al. (2021) [102]	Lentivirus	DMEM, KSR	LIF	MSC Medium: KO DMEM, FBS, SB 431542, NEAAs, l-glutamine	Mesenchymal stromal cells		Inflammatory disorders
Gasson et al. (2021) [104]	Sendai virus	DMEM/F12, KSR	bFGF	MSC Medium: DMEM, KSR, SB 431542, NEAAs, l-glutamine	Mesenchymal stromal cells		Inflammatory disorders
				Osteogenesis Induction: α-MEM, FBS, b-glycerophosphate, ascorbate-2-phosphate, Dexamethasone, BMP-2		Osteocytes	
Whitworth et al. (2014) [20]	Lentivirus	DMEM, KSR	LIF	MSC Medium: DMEM, KSR, SB 431542, NEAAs, l-glutamine	Mesenchymal stromal cells		Osteoarthritis
				Adipogenesis Differentiation Kit		Adipocytes	
				Chondrogenesis Differentiation Kit + Hydrogel		Chondrocytes	
				Osteogenesis Differentiation Kit + Hydrogel		Osteocytes	

DMEM, Dulbecco's modified Eagle's medium; F12, Ham's F-12 Nutrient Mix; FBS, fetal bovine serum; EGF, epidermal growth factor; KO, knockout; KSR, knockout serum replacement; NEAA, nonessential amino acid; IMDM, Iscove's modified Dulbecco's medium; MiP, mesodermal iPSC-derived progenitor cell; RPMI, Roswell Park Memorial Institute-1640 medium; TGF-β1, transforming growth factor beta 1; VEGF, vascular endothelial growth factor; MSC, mesenchymal stem cell; α-MEM, α-minimum essential medium; BMP-2, bone morphogenic protein 2.

### Differentiation capacity of ciPSCs: tissue and organ regeneration

To determine the clinical utility of ciPSCs for tissue regeneration, it is important to investigate the viability of the cell lineages they may produce, and whether treatment can improve patient outcomes compared to current therapies. In a recent pilot study by Chow et al. (2020), ciPSCs were differentiated into neural progenitor cells (NPCs) to treat two dogs with spinal cord injuries, but no significant clinical or electrophysiological improvement was observed [94]. These results could have been due to a limited sample size or that the injection of cells was done without a protective matrix [94]. Foster et al. (2018) showed that Shear Thinning Hydrogel for Injectable Encapsulation and Long-term Delivery (SHIELD) provides mechanical protection for the membrane of iPSCs during injection into affected tissues and may be used to mitigate the shear forces on ciPSCs associated with injection [94,97].

A similar study to Chow et al. (2020) by Chandrasekaran et al. (2021) differentiated ciPSC-like cells into NPCs that were used to repair damaged nervous tissue in dogs [93]. They found that despite incomplete reprogramming of ciPSCs, canine NPCs had reduced axonal fasciculation and shorter neuritis, demonstrating lower mature neuronal differentiation efficiency than human NPCs [93]. NPCs in both studies were produced by following a similar protocol using ciPSC culture media containing bFGF and epidermal growth factor (EGF) but differing in the composition of the neural induction media as summarized in Table 3.

Cardiac cell lineages on thin films of functionalized carbon nanotubes (CNTs) were generated from ciPSCs by Mondal et al. (2022) and Natarajan et al. (2021) after EB formation [95,96]. In both studies, the hanging drop technique was used to form EBs, in which the ciPSC cell suspension was pipetted onto the lid of a culture dish, allowing EBs to form through aggregation at the bottom of the drop [98,99]. The resulting cardiomyocytes exhibited cardiac markers, including *AMHC*, *NKX2.5*, and *TBX5*, and had good differentiation efficiency on CNT films compared to standard feeder culture [95,96].

Canine mesodermal iPSC-derived progenitor cells (MiPs) were formed from ciPSCs by Quattrocelli et al. (2015), which can differentiate into any mesodermal lineage [16]. Canine fibroblast and mesoangioblast iPSCs were cultured on feeder layers of MEFs in hiPSC medium containing bFGF, then induced to form EBs in suspension culture [16]. Canine MiPs were differentiated from EBs using continuous passaging and fluorescence-activated cell sorting and subsequently differentiated into cardiomyogenic, skeletal myogenic, osteogenic, adipogenic, smooth muscle, and endothelial tissues in vitro (Table 3). Canine MiPs were also injected into hindlimb skeletal muscles and the myocardium of immunodeficient dystrophic mice, resulting in successful engraftment, functional regeneration of both striated muscle types, and improved regenerative outcomes [16].

Endothelial cells (ECs) were derived from ciPSCs by Lee et al. (2011) and were maintained in culture with LIF and bFGF. EBs were formed after the removal of LIF/bFGF, and cells were sorted based on the presence of the endothelial marker, CD31 [22]. After injection, ciPSC-derived ECs significantly improved revascularization and cardiac contractility in mice with hindlimb ischemia and myocardial infarction, respectively [22]. Nishimura et al. (2013) established a novel protocol for platelet production from ciPSCs, in which a coculture system with VEGF formed ciPSC-derived sacs that produced platelet-releasing mature megakaryocytes [38]. These platelets were similar in morphology and binding capability to canine peripheral platelets [38].

Although hiPSCs are pluripotent, they have been shown to have a propensity to differentiate into their parental cell lineage, likely due to epigenetic memory [100,101]. Therefore, selection of iPSC lines derived from specific cell types is an important consideration in developing regenerative therapies. From a clinical standpoint, epigenetic memory could be leveraged in iPSC treatments to increase the likelihood of differentiation toward a desired cell lineage or reduce the chances of aberrant development [101]; however, this may hinder the acquisition of true pluripotency in research contexts. To date, no studies have compared differences in differentiation tendencies among ciPSC lines, which is a necessary consideration for developing efficient tissue engineering strategies and safe ciPSC-based cytotherapies.

Taken together, evaluating the differentiation capacity of ciPSCs into specific cell lineages and transplantation into damaged tissues is an important step toward their use in regenerative medicine. To date, studies have investigated ciPSC therapy for canine neurodegenerative, hematopoietic, musculoskeletal, cardiovascular, and platelet disorders. ciPSCs can also be differentiated into other stem cells such as MSCs, which have been used clinically for the various trophic factors and immunomodulatory cytokines that make up their secretome [102].

### ciPSC-derived MSCs

Canine MSCs (cMSCs) are immunomodulatory and, thus, may serve as a good treatment option for dogs with inflammatory diseases, including osteoarthritis, inflammatory bowel disease, and autoimmune diseases [103]. Functionally, cMSCs have CXCR-4, which is a critical regulator of mobilizing MSCs to sites of inflammation, and constitutively express immunomodulatory factors, including iNOS, GAL-9, *TGFβ1*, *PTGER2A*, and *VEGF*, along with the pro-inflammatory mediators *COX-2*, *IL1β*, and *IL-8* [103]. As multipotent cells, cMSCs can further differentiate into specific cell types for tissue and organ regeneration. The unlimited passage potential of ciPSC-derived cMSCs (ciMSCs) is advantageous for clinical use, especially given that the cell donor may also be the recipient [104].

A few studies have demonstrated that ciPSCs can successfully differentiate into ciMSCs, confirming the mesenchymal phenotype through trilineage differentiation by either teratoma or embryoid body formation and identifying surface markers, including CD44, CD29, and CD90 [20,27,37,103,105]. Differentiation protocols were consistent across all studies, in which the MSC culture medium consisted of DMEM, FBS, SB 431542, nonessential amino acids, and l-glutamine (Table 3). SB 431542 is a TGFβ/Activin inhibitor that prevents pluripotent gene expression in ciMSCs [106].

Compared to conventional MSCs directly harvested from the donor adipose or bone marrow, ciMSCs have been shown to have equivalent in vitro immunomodulatory properties, including suppression of T cell proliferation, IFNG production, and IFNG release from activated PBMCs [20,27]. However, on a molecular level, ciMSCs had greater expression of immunomodulatory factors, including Th-2 related or immune suppressive cytokines relative to conventional MSCs [27]. Importantly, ciMSCs injected systemically into adult dogs did not form tumors, even after a 1-year follow-up [27]. A lack of tumor formation following systemic injection is of particular clinical relevance, given that it is the most common route for administering MSCs to treat inflammatory disorders [27]. In addition, ciMSCs have been shown to proliferate at a much higher rate than conventional MSCs, which appears to be an inherent and advantageous characteristic in terms of treatment availability [27].

ciMSCs also display phenotypic stability and equivalent differentiation capacity to conventional MSCs following environmental stimuli. When maintained in the right inductive media, ciMSCs can be differentiated into osteogenic, chondrogenic, and adipogenic cells [20,27,105]. For example, ciMSCs can be differentiated toward a chondrogenic-specific lineage when added to hydrogels containing pentosan polysulfate and hyaluronic acid [20]. Like cMSCs, ciMSCs responded well to osteogenic TFs such as BMP-2 by effectively differentiating into osteogenic cells [103,107–110].

In summary, ciMSCs may be applied clinically to modulate inflammation or for more specific tissue regeneration in canines compared to ciPSCs. Current research into ciPSC applications is focused on deriving specific cell and tissue types. Given that most individual articles focus on a single pathology, more research is required to confirm clinical outcomes following treatment with ciMSCs for comorbid conditions.

### Disease modeling and drug delivery systems

Another promising application of ciPSCs is their use in disease modeling and drug delivery screening. ciPSCs can be produced from dogs with specific diseases or genetic abnormalities and can maintain the aneuploid karyotype of parental cells [21]. These cells can be used to model diseases in vitro or be manipulated with gene correction strategies, allowing for retransplantation. An excellent example of disease modeling is iPSC-derived 3D organoids to assess cell-to-cell interactions, disease progression, and treatment delivery in vivo. This technology has mainly been studied using human iPSCs to model organ systems [98,99] such as hiPSC-derived kidney organoids, which demonstrated nephron toxicity from tacrolimus treatment [110]. Animal models have also been used to test the safety and clinical benefit of hiPSC-derived organoids such as the transplantation of hiPSC-derived liver organoids into piglets [111]. To date, no studies have reported production of ciPSC-derived organoids. Future research could investigate the feasibility of ciPSC-derived organoids for understanding the etiology and underlying pathology of canine conditions.

## Other Barriers to ciPSC Applications

In addition to the reprogramming barriers discussed above, the use of ciPSCs in clinical settings is largely limited by their safety profile and cost to produce. The potential for both immunological risks and tumorigenesis are the main safety risks associated with ciPSC treatments. Given the high costs of producing ciPSCs, it is important to also reflect on the cost, benefit, and risk of their use relative to current treatment options.

### Tumorigenesis and immunological responses

A major safety concern associated with the clinical use of ciPSCs is oncogenesis following transgene reexpression. The risk of tumorigenesis following ciPSC transplantation is due to the highly proliferative nature of ciPSCs and difficulties in complete silencing of transgenes. The Yamanaka factor and proto-oncogene *c-MYC* plays a key role in self-renewal and metabolic regulation during iPSC generation and normal embryonic development, but once transplanted, this TF may become tumorigenic if it continues to be expressed [112,113]. In fact, the aberrant expression of *c-MYC* has been observed in ∼70% of cancers [114]. However, MYC proteins are important regulators of cell growth, making other members of this protein family strong candidates for the replacement of c-MYC.

L-MYC has been used as a replacement reprogramming factor for c-MYC when generating ciPSCs [115] and has been shown to promote self-renewal but not tumorigenesis in canine fetal fibroblasts [115]. GLIS1 is another viable alternative to the oncogenic c-MYC and was used by Kim et al. (2020) to generate ciPSCs [24]. To mitigate oncogenic concerns, GLIS1 has been shown to increase the genome stability of iPSCs [78]. GLIS1 was also found to directly reprogram somatic cells into iPSCs more fully than c-MYC, while suppressing the proliferation of colonies of incompletely reprogrammed cells [24]. Furthermore, the capacity of GLIS1 to reprogram cells appears independent of cell type and methodology, making it a strong candidate for generating clinically ready ciPSCs [116,117].

Aside from transgene reexpression, in vitro generation, expansion, and differentiation of ciPSCs are labor intensive and have the potential to select for epigenetic aberrations and/or genetic mutations that may promote oncogenesis [118–120]. Genomic and epigenomic instability in iPSCs may result from artificial culture adaptation, making optimized reprogramming and differentiation methods of significant value in generating clinically safe ciPSCs. Researchers have also investigated a cellular “suicide system” to protect against tumorigenesis. In mice, iPSCs expressing “suicide genes” underwent programmed cell death weeks after transplantation upon exposure to the prodrug, ganciclovir [121]. This system acts as a fail-safe in the event of aberrant growth or modification and is particularly useful when using integrating viral vectors [31,93,122]. Another method discovered in mouse models is the activation of tumor-suppressor alternative reading frames in iPSC-derivatives [123]. It is possible that one or a combination of these methodologies may be able to eliminate the potential of tumor formation following implantation of ciPSCs.

Immunological rejection of allogeneic ciPSC transplants is another major barrier to their clinical application. The use of autologous cells can mitigate the risk of immune rejection [22]. While commercialization of ciPSC-based therapies such as autologous stem cell therapy is a safer option for recipients, this may be an unrealistic solution due to the high cost of individual ciPSC line production [24,124].

As a solution to immunological rejection associated with allogenic iPSC treatment, research is exploring immune modification of iPSCs so that their derivatives are unrecognizable by the recipient's immune system [125]. Strategies have primarily been investigated in mice and humans and include immune cloaking [126,127], HLA and MHC alteration [128], and expression of immunomodulatory transgenes [126]. A promising method to reduce the immunogenicity of ciPSCs by avoiding T cell recognition may be eliminating antigen-presenting functions through HLA depletion. To date, research has mainly focused on reducing the expression of HLA class 1 by targeting the beta-2 microglobulin (B2M) chain, which is a conserved subunit required for surface expression of all HLA class 1 antigen heterodimers [129]. Technologies used for HLA depletion may include RNA interference (RNAi) with small interfering RNAs (siRNAs) or short hairpin RNAs (shRNAs) for post-transcriptional regulation or transcription activator-like effector nucleases (TALENs), CRISPR-Cas9, or vector-mediated gene targeting technology for complete depletion [129].

Interestingly, reports suggest that mismatches at the HLA DR locus result in the most potent alloimmune response against transplanted organs such as heart, kidney, and lung [130–133], potentially making this a stronger candidate than HLA class 1 for immunogenic manipulation in iPSCs. A study by Kwon et al. (2021) demonstrated the utility of TALENs to selectively eliminate HLA DR expression in human iPSCs [130]. Subsequent differentiation of HLA DR knockout iPSCs into dendritic cells (DCs)—potent activators of both innate and adaptive immunities through antigen presentation—showed no activation of allogenic T cells compared to wild-type DCs [130]. Molecular tools shown to successfully manipulate the antigen presenting functions of human and mice iPSCs and cellular derivatives could be used on ciPSCs to test the feasibility of generating safe “universal” treatments for organ and tissue regeneration.

Culture conditions of ciPSCs are another risk factor that may influence the potential for immunological rejection or pathogen contamination. Currently, most ciPSC cultures are maintained in chemically undefined conditions, in the presence of animal products like FBS or KSRM, and on feeder cells such as MEFs, which may increase the risk of immune rejection by modifying cell surface components in ciPSCs [18,134]. Preparing MEFs requires a considerable amount of labor and increases the risk of contaminating ciPSCs with foreign agents, making xeno-free or feeder-free alternatives a necessary clinical safety measure for developing ciPSC treatments. Most feeder-cell alternatives have been investigated for culturing hiPSCs and include combinations of bioactive molecules such as adhesion and extracellular matrix (ECM) proteins or synthetically derived peptides and polymers [135].

Cadherins are adhesion molecules that regulate proliferation, morphogenesis, and differentiation of stem cells, making them a viable substrate to support feeder-free culture. In particular, E-cadherin is highly expressed in ESCs and mediates iPSC adhesion and colony formation [136,137]. ECM proteins such as laminin and fibronectin have been investigated as substrates for feeder-free culture, both of which appear to promote the pluripotency of hESCs and hiPSCs in feeder-free conditions [138,139].

Synthetic proteins for culturing iPSCs may circumvent both immunogenic and pathogenic risks associated with animal-derived proteins. A study by Melkoumian et al. (2010) demonstrated that a synthetic peptide acrylate surface conjugated to ECM proteins was able to support the growth of multiple hiPSC lines, which maintained an undifferentiated phenotype, normal karyotype, and were able to generate teratomas in vivo after being cultured in xeno-free conditions [140]. Synthetic polymers may provide physiologically relevant surfaces for long-term, feeder-free iPSC culture. Results from Villa-Diaz et al. (2010) demonstrated that hESCs cultured on poly[2-(methacryloyloxy)ethyldimethyl-(3-sulfopropyl)ammonium hydroxide] in feeder-free conditions retained pluripotency and a normal karyotype after 25 passages; pluripotency was validated through teratoma formation in vivo [141].

Although the majority of feeder-free substrates have been tested in hiPSCs, Kimura et al. (2020) showed that ciPSCs maintained on the feeder-free substrate iMatrix-511 with the chemically defined stem cell culture media StemFit AK02N successfully retained pluripotency and a normal karyotype [18]. These results suggest that StemFit AK02N and iMatrix-511 culture systems may mitigate concerns related to xenogeneic products used in conventional ciPSC production systems (Fig. 1). Future research into the viability of xeno-free alternatives to feeder cells and culture media for maintaining ciPSCs may benefit from applying strategies used in hiPSC culture, some of which may include technical modifications to protocols reviewed in Vinuelas et al. (2021) [142].

## Conclusions and Perspectives

The potential utility of ciPSCs in both human and veterinary regenerative medicine warrants further research into improving their production. It appears that canine somatic cells have a larger epigenetic barrier to pluripotency acquisition than other mammals, which may explain frequent reports of incomplete SCR in canine. The unique, dynamic epigenetic landscape of chromatin in canine somatic cells presents novel targets for improving the efficacy of SCR using more than the typical OSKM factors, SMCs, selective mutation of pluripotency factors, and gene-editing technologies. Given that multiple factors dictate the successful attainment of pluripotency in canine cells, solutions should be studied both individually and in combination.

Standardized reproducible protocols for ciPSC generation that considers species-specific differences in SCR may avoid inconsistent efficiencies and allow for more robust comparative research. Conserved and divergent pluripotent gene expression patterns have been documented across various species [90]. Utilizing canine-specific genes associated with pluripotency may result in *bona fide* ciPSCs that can be used in developing novel pluripotent stem cell-based therapies in the dog.

Finally, defining the final pluripotent state of ciPSCs may inform better media choices for both activating and maintaining pluripotency in canine cells. However, the final pluripotency state of ciPSCs remains elusive and should be studied considering the “rosette state” of pluripotency.

To ready ciPSCs for clinical applications, attention must be directed to optimizing culture conditions, reprogramming, and differentiation protocols with the intent of improving safety and long-term cell viability. Insights can be gained from reports of successful iPSC generation in human, murine, and large domesticated animals. The potential use of ciPSCs for autologous regenerative therapy and disease modeling are the primary justifications for their high production costs. However, their use is primarily limited by ineffective SCR, inconsistent production efficiencies, and safety concerns. With future research efforts into ciPSCs, we may progress one step closer to revolutionizing the treatment of many canine diseases.
